# Barium Aspiration in an Infant: A Case Report and Review of Management

**DOI:** 10.3389/fped.2014.00037

**Published:** 2014-05-01

**Authors:** M. Jackson, N. Kapur, V. Goyal, K. Choo, A. Sarikwal, I. B. Masters, Alan F. Isles

**Affiliations:** ^1^Department of Respiratory Medicine, Royal Children’s Hospital, Queensland Children’s Respiratory Centre, Brisbane, QLD, Australia; ^2^University of Queensland, Brisbane, QLD, Australia; ^3^The Queensland Children’s Medical Research Institute, Brisbane, QLD, Australia; ^4^Department of Paediatric Surgery, Royal Children’s Hospital, Brisbane, QLD, Australia; ^5^Department of Medical Imaging, Royal Children’s Hospital, Brisbane, QLD, Australia

**Keywords:** barium, aspiration, tracheo-esophageal cleft, tracheo-esophageal fistula

## Abstract

We describe a case of bilateral inhalation of barium in an infant following a barium swallow for investigation of dusky spells associated with feeds. A bronchoscopy subsequently revealed the presence of a mid-tracheal tracheo-esophageal cleft. To date, little has been reported on barium aspiration in children and there is no consensus for management. We review the literature on barium aspiration, its consequences, and make recommendations for management.

In any infant with respiratory symptoms that occur contemporaneously with feeds, aspiration through a laryngeal cleft, tracheo-laryngeal cleft, tracheo-esophageal fistula, and severe gastro-esophageal reflux should all be considered in the differential diagnosis (1). Radiological imaging techniques may not be able to differentiate primary from secondary aspiration, especially in the very young nor separate primary aspiration from high structural lesions such as H-type fistula (2).

We report a case of an isolated mid-tracheal tracheo-esophageal cleft in an infant where management was complicated by inhalation of barium into the lungs during a radiographic contrast procedure.

## Case Report

A 6-day-old infant presented to the emergency room (ER) of our tertiary care hospital with tachypnea, recession, and dusky episodes associated with feeding. He was moderately dehydrated and lethargic at presentation with a weight loss of 390g from his birth weight of 2990 g. There was no cough or vomiting associated with these episodes. He was afebrile but tracheal tug and lower costal retractions were present. Auscultation of his chest was normal. In the ER, his oxygen saturation was 70% and 0.8 L/min of oxygen was commenced with normalization of oxygenation. He was subsequently found to consistently desaturate to around 80% during feeds. These episodes of desaturations were not associated with cough.

**Figure 1:**
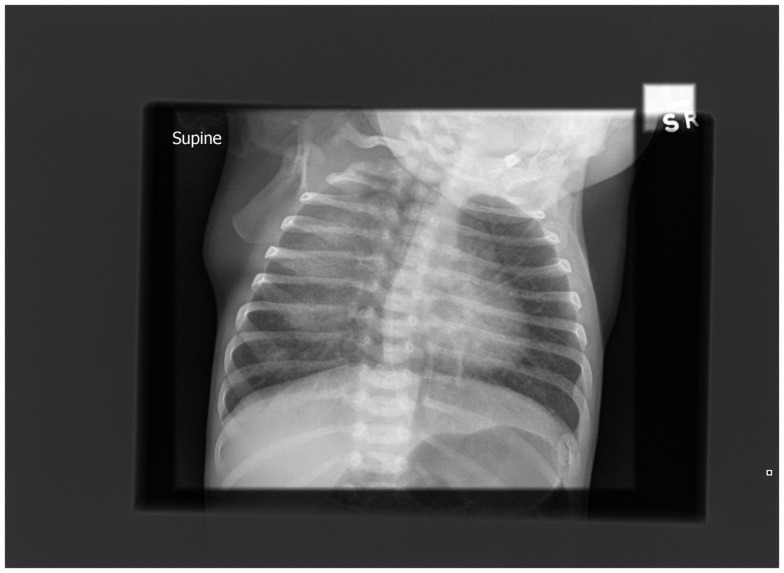
**Chest radiograph on admission showing right-sided changes**.

**Figure 2:**
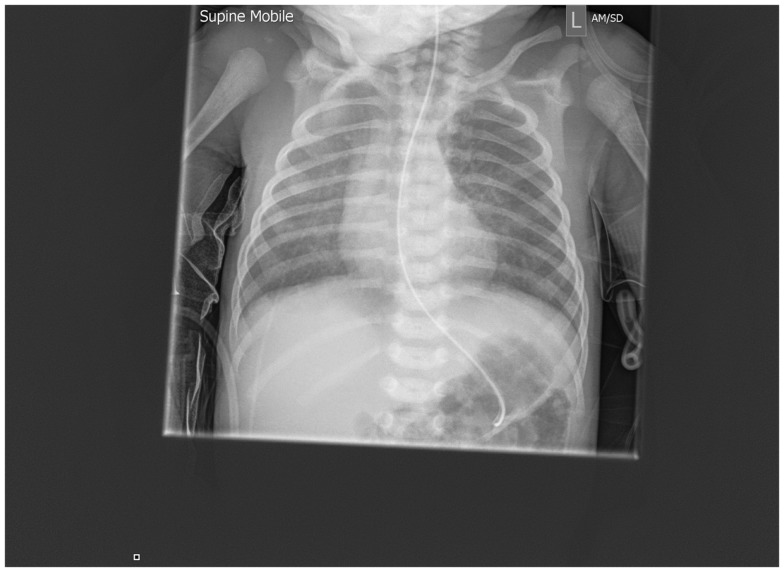
**Repeat chest radiograph 1 day after admission demonstrating resolution of the changes within 24 h**.

A chest radiograph (Figure 1) showed diffuse and homogenous airspace opacity in the right upper and middle zones with prominent perihilar markings. Following a septic screen, he was commenced on intravenous (IV) antibiotics and IV fluids. The septic screen was subsequently negative. The right-sided chest opacity had cleared by the following day (Figure 2) perhaps reflecting resolution of mucus plugging. The working diagnosis at the time was aspiration disease. Because of suspected gastro-esophageal reflux, a nasogastric tube was inserted and the baby received continuous feeds via the tube and did not have any further desaturations.

**Figure 3:**
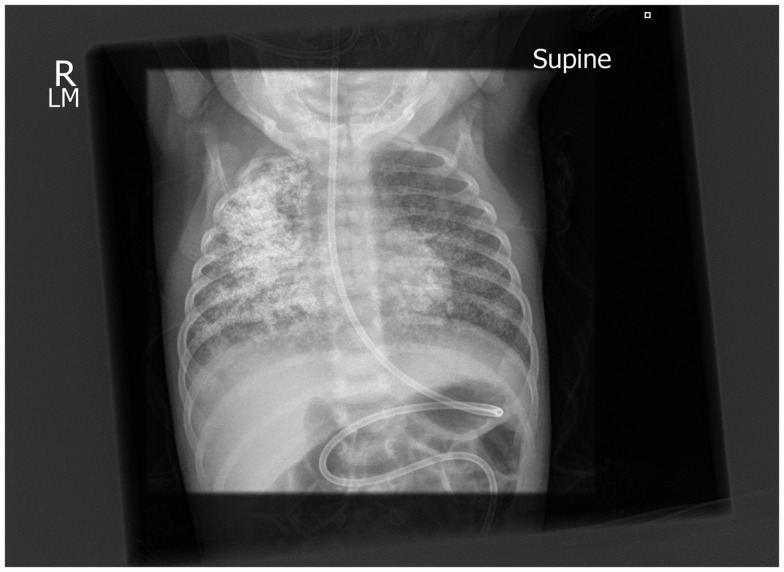
**Post-aspiration chest x-ray showing well-defined radio-dense opacities through all zones of the right lung and similar but less extensive changes on the right**.

A barium esophagram using a 50% dilution of 125 w/v barium for suspected gastro-esophageal reflux was performed via the nasogastric tube with the infant in the supine position and showed prompt reflux back to the cervical esophagus with significant aspiration of the contrast into the trachea and the lungs bilaterally (Figure 3). The baby did not become acutely distressed during the study with oxygen saturations being 98% pre-study and 96% post-study. The nasogastric tube was replaced with a trans-pyloric tube for feeding.

**Figure 4:**
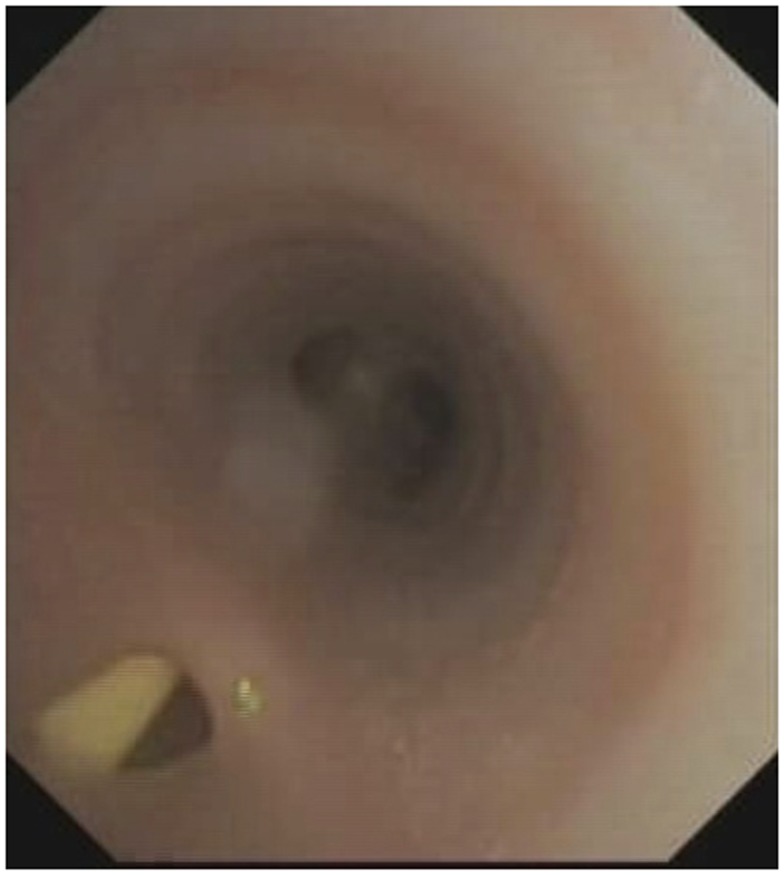
**Tracheoesophageal cleft with nasojejunal tube visible in the esophagus**. Note the absence of a “fistula” or “tube connection” but a confluence of the tracheal and esophageal lumens.

Due to the significant aspiration seen on the study, the Respiratory Service was consulted and a flexible bronchoscopy was performed to exclude congenital structural abnormalities. At bronchoscopy, a large tracheo-esophageal cleft approximately 3.8 mm in diameter was seen on the posterior wall of the trachea 4 cm above the carina with the nasojejunal tube in the esophagus clearly visible through the cleft (Figure 4). The remainder of the airway was structurally normal. A bronchitis appearance was present.

Surgical repair of the fistula was deferred initially due to the concerns around the significant contrast material in the lungs and a preference to achieve weight gain prior to repair. Surgical repair through a cervical incision was eventually undertaken on day 42 of life. The fistula was cannulated bronchoscopically from the tracheal side with a guide-wire fed into the esophagus. This was then advanced into the stomach from where it was retrieved and brought out through the mouth. This enabled a right-sided cervical approach to the fistula/cleft where the esophageal and tracheal ends of the fistula/cleft were surgically identified and over-sewn. The sternocleidomastoid muscle on the right was mobilized and used as a patch on the esophagus.

The baby failed extubation on day 3 post-surgery at 3 h due to severe upper airway obstruction manifesting as inspiratory stridor, marked intercostal recession and sternal retraction, absence of cry, and an altered sound to his cough. He was re-intubated. A bronchoscopy at day 16 post-surgery revealed vocal cord immobility with failure of abduction presumed to be due to a traction-induced neuropraxia of the recurrent laryngeal nerve. Post-bronchoscopy, he was extubated successfully to high-flow nasal oxygen, which was subsequently weaned. Nasojejunal tube feeding was continued because of the vocal cord dysfunction. The stridor and hoarse cry slowly improved over a 2-week period with conservative management. On day 77 of life, a videofluoroscopy showed no evidence of laryngeal penetration so breast feeds were resumed and the nasojejunal tube was removed on day 82. The baby was transitioned to full breast feeds and discharged. Despite the barium visible on the chest radiograph, there was no respiratory distress or requirement for supplemental oxygen.

## Discussion

Barium aspiration is typically accidental. When it does occur, chest radiographs and high-resolution CT examinations post-aspiration produce striking images because of the high atomic number of barium (z 56) with an associated high absorption of x-rays producing a “white-out” appearance.

Aspiration of minor amounts of barium during diagnostic procedures is reportedly common and appears not to be clinically significant. Aspiration of significant amounts of barium in infants is rare and there is no consensus in the literature on how to manage such aspiration. Importantly, there is no clear consensus as to the effect of inhaled barium on the lungs.

This case illustrates two key points. Firstly, it highlights the limitations of imaging techniques in differentiating primary and secondary aspiration especially in the very young. While the fistula was large in this case (3.8 mm), small H-type fistulas can be difficult to identify on contrast studies. A negative contrast study does not exclude the diagnosis of a fistula and direct visualization by bronchoscopy should be strongly considered if there is a consistent history of respiratory symptoms related to feeds. Visualization can be with either a rigid or flexible bronchoscope. Which ever instrument is used, the key points are that fistulae are usually higher in the trachea than expected and significant skill is required to identify a fistula as the opening of the fistula on the posterior wall of the trachea may be very small or occluded with mucus. Small H-type fistulae can be easily missed.

Obtaining guidance from the literature to predict the clinical course and plan the management of our patient proved disappointing. There has been no substantial case series to provide an evidence-based approach to treatment of barium aspiration in children. This is perhaps to be expected when significant barium aspiration is an uncommon event. Review of available literature involved searching electronic databases including EMBASE, MEDLINE, and PUBMED. Only articles written in English were considered. The search strategy included searching for the following terms separately: baritosis; barium aspiration; contrast aspiration; tracheo-esophageal cleft, and barium bronchography.

The consequences of aspiration of barium are varied and influenced by the patient’s age, pre-existing clinical state, the concentration of the barium used (3, 4), the volume aspirated, and concomitant aspiration of gastric contents (5). The distribution pattern of the barium within the tracheo-bronchial tree and lung is, in turn, determined by the subject’s posture at the time of the study and clearance mechanisms such as cough, mucociliary escalator clearance, and cellular ingestion.

Unlike our patient who demonstrated no acute signs or symptoms, other case reports, largely in adults, describe acute respiratory distress, pneumonitis, sepsis, and even death (6, 7). It is difficult in many of these case reports to separate out the effect on the lung of the barium from that of the aspirated gastric contents. Since our patient was still a neonate, the gastric contents are less likely to be acidic and thus less likely to cause aspiration-related lung injury (8). Lopez-Castilla et al. described a 2-month-old child with gastro-esophageal reflux who developed acute respiratory distress and an oxygen requirement after aspirating barium following a contrast study (3). Ten fiberoptic bronchoalveolar lavages were undertaken in an endeavor to remove the barium. The authors stated that barium was recovered but the amount was not quantified. A CT scan of the chest 4 months after the episode still showed significant residual barium and micro-nodular densities. Despite this, the authors concluded that therapeutic bronchoalveolar lavage was mandatory after barium aspiration. Wani and Yeola, in a case report of barium aspiration in an adult, took the contrary view and recommended against bronchoalveolar lavage arguing that it may disseminate the barium further within the bronchoalveolar system (9).

Case reports about the long-term effects of barium aspiration provide varying information. Small amounts of barium are usually well-tolerated in the bronchial tree. Once aspirated, the barium particles that are not coughed out or removed by mucociliary clearance accumulate in alveolar spaces (10). Voloudaki et al., using high-resolution CT scans, concluded that the barium particles are likely to be phagocytosed by alveolar macrophages and can potentially cause interstitial fibrosis by crossing into the alveolar or peribronchial interstitial tissue (11). These authors reported thickening of interlobular septae, sub-pleural cysts, and centrilobular micro-nodules along with barium particles in a sub-pleural distribution in an adult 1 year after barium inhalation. They concluded that barium is capable of producing clinically mild, silent fibrosis. Venkatraman et al. reported peribronchial interstitial changes after barium aspiration (12). By contrast, Marchiori et al. described baritosis in which inhaled particulate matter lies in the lungs for years without producing symptoms, interference with lung function, or liability to develop pulmonary or bronchial infections or other thoracic disease (13).

Prior to the introduction of high-resolution CT scans for the diagnosis of bronchiectasis, barium, and even oil contrast bronchograms were frequently performed. Wilson et al. reported that barium bronchography in dogs resulted in a mild transient inflammatory reaction, which was quickly replaced by a bland foreign body reaction (14). They also reported on 16 cases of barium bronchography in humans and found no ill-effects, either acute or chronic. They concluded that barium sulfate in the lung behaves as a relatively inert foreign body (14). Shook and Felson described 19 cases, including 3 children, in which barium was atomized into the lung for the purpose of bronchography (15). Teixeria and Texieria reported on over 200 human barium bronchograms without a single adverse event (16). Nelson et al. reported on an invaluable study in which 89 patients underwent barium sulfate bronchography at intervals of up to 6 months before lung resection (17). There was no evidence of histologic fibrotic pulmonary changes for up to 6 months after the bronchograms.

Further evidence of the rather benign nature of barium in the lung comes from Doig who described nine cases of baritosis in factory workers exposed to barium dust (18). Based principally on lung function tests, he described baritosis as a benign pneumoconiosis. He reported partial clearing of the radiographic changes over 9 years after exposure ceased.

## Conclusion

In pediatrics, imaging techniques may not differentiate primary from secondary aspiration. Factors that are likely to influence outcomes post-aspiration are the child’s pre-existing clinical state, the volume, and concentration of the barium sulfate used as well as the immediate post-aspiration clinical status. If barium is aspirated, we believe that the evidence favors supportive care in most cases, with therapeutic bronchoalveolar lavage to be considered only in cases with significant respiratory symptomatology. The evidence suggests that the barium will remain in the lung for an extended time, be relatively inert and that the risk of fibrosis or other complications remains low. We plan to follow and report on his clinical and radiological progress.

## Conflict of Interest Statement

The authors declare that the research was conducted in the absence of any commercial or financial relationships that could be construed as a potential conflict of interest.
